# Environmental Estrogens Induce Mast Cell Degranulation and Enhance IgE-Mediated Release of Allergic Mediators

**DOI:** 10.1289/ehp.9378

**Published:** 2006-10-03

**Authors:** Shin-ichiro Narita, Randall M. Goldblum, Cheryl S. Watson, Edward G. Brooks, D. Mark Estes, Edward M. Curran, Terumi Midoro-Horiuti

**Affiliations:** 1 Department of Pediatrics, Child Health Research Center and; 2 Department of Biochemistry and Molecular Biology, University of Texas Medical Branch, Galveston, Texas, USA

**Keywords:** allergy, asthma, β-hexosaminidase, environmental estrogen, estradiol, estrogen receptor α, IgE, mast cells

## Abstract

**Background:**

Prevalence and morbidity of allergic diseases have increased over the last decades. Based on the recently recognized differences in asthma prevalence between the sexes, we have examined the effect of endogenous estrogens on a key element of the allergic response. Some lipophilic pollutants have estrogen-like activities and are termed environmental estrogens. These pollutants tend to degrade slowly in the environment and to bioaccumulate and bioconcentrate in the food chain; they also have long biological half-lives.

**Objectives:**

Our goal in this study was to identify possible pathogenic roles for environmental estrogens in the development of allergic diseases.

**Methods:**

We screened a number of environmental estrogens for their ability to modulate the release of allergic mediators from mast cells. We incubated a human mast cell line and primary mast cell cultures derived from bone marrow of wild type and estrogen receptor α (ER-α )–deficient mice with environmental estrogens with and without estradiol or IgE and allergens. We assessed degranulation of mast cells by quantifying the release of β -hexosaminidase.

**Results:**

All of the environmental estrogens tested caused rapid, dose-related release of β -hexosaminidase from mast cells and enhanced IgE-mediated release. The combination of physiologic concentrations of 17β -estradiol and several concentrations of environmental estrogens had additive effects on mast cell degranulation. Comparison of bone marrow mast cells from ER-α –sufficient and ER-α –deficient mice indicated that much of the effect of environmental estrogens was mediated by ER-α .

**Conclusions:**

Our findings suggest that estrogenic environmental pollutants might promote allergic diseases by inducing and enhancing mast cell degranulation by physiologic estrogens and exposure to allergens.

The prevalence and morbidity of asthma and other allergic diseases have increased dramatically during the last 30 years, particularly in industrial countries (Burr et al. 2006). The onset of asthma most commonly occurs in early childhood (Yunginger et al. 1992). Asthma is more common in males during infancy, childhood, and preadolescence (Yunginger et al. 1992). However, from late adolescence to middle age, females have a higher prevalence and morbidity from asthma (De Marco et al. 2002; Yunginger et al. 1992). Although the increase in overall prevalence and the cause of these pattern differences between the sexes are not well understood, we questioned whether female gonadal hormones and their mimetics might be involved.

We recently found that physiologic concentrations of estradiol (E_2_) rapidly stimulate murine and human mast cell lines (RBL-2H3 and HMC-1) and primary cultures of bone marrow–derived mast cells (BMMC) to release β -hexosaminadase (β -hex), a marker for the granules that contain preformed allergic mediators (Zaitsu et al. 2006). These low doses of E_2_ also enhanced the synthesis and release of leukotriene C_4_ (LTC_4_) by RBL-2H3 cells. In addition to these direct effects, E_2_ potentiated IgE-dependent synthesis and release of β -hex, and particularly LTC_4_. The finding that the estrogen receptor (ER) antagonists tamoxifen and ICI 182,780 inhibited these effects suggested that these estrogenic effects were mediated through specific ERs (ER-α or ER-β ). This proposition was substantiated by demonstrating that BMMCs derived from ER-α knockout (KO) mice did not degranulate in response to E_2_. We also analyzed the expression of ER-α and ER-β by reverse transcriptase-polymerase chain reaction and could detect only ER-α on RBL-2H3, HMC-1, and BMMCs (Zaitsu et al. 2006). Another recent study provided evidence for estrogen effects on allergic sensitization/reactions by showing a relationship between an ER-α gene (*ESR1*) polymorphism and airway hypersensitivity, and an age-related decline in lung function in females with asthma (Dijkstra et al. 2006).

Estrogens and other steroid hormones use two different major cellular pathways to exert their regulatory effects. One pathway is via genomic receptors acting as transcription factors on gene expression. However, an alternative pathway acting via plasma membrane receptors is more often involved in the rapid effects of steroids occurring within seconds to minutes (Watson et al. 1999; Watson and Gametchu 2003). This nongenomic pathway is involved in secretory responses to both physiologic and nonphysiologic estrogens (Bulayeva et al. 2005).

Many environmental pollutants have estrogen-like activities and thus are termed environmental estrogens or xenoestrogens (Newbold et al. 2006; Wozniak et al. 2005). These components can be involved in both genomic and nongenomic pathways of estrogen action, but have recently been shown to be very potent when acting via the non-genomic pathway (Wozniak et al. 2005), although they are very weak activators of the genomic pathway. If environmental estrogens act at such low levels, then the widespread presence of these compounds in our environment are of concern as causes for the increasing prevalence of diseases such as asthma.

Examples of environmental estrogens include the dioxins, dichlorodiphenyl-trichloroethane (DDT) and its metabolite dichlorodiphenylethylene (DDE), hexachloro-cyclohexane, polychlorinated biphenyls (PCBs), and alkylphenols and their derivatives (nonylphenol, octylphenol, bisphenol A). The most common source of these pollutants is through contaminated water and foods (Aravindakshan et al. 2004; Falconer et al. 2006). We therefore questioned whether environmental estrogens could have effects on allergic sensitization and clinically relevant reactions, such as for asthma. The goal of the present study was to identify possible mechanisms by which environmental estrogens, alone or in combination with endogenous estrogens, might promote the development of allergic diseases.

## Materials and Methods

### Cells and cell culture

We obtained the HMC-1 human mast cell line from J.H. Butterfield (Mayo Clinic, Rochester, MN) (Butterfield et al. 1988). Cells were cultured in Iscove's modified Dulbecco's medium (IMDM; Cellgro, Kansas City, MO) with 10% iron-supplemented calf serum (Hyclone, South Logan, UT). To avoid exposure to estrogens during culture, we used steroid-stripped fetal calf serum (FCS) and phenol red–free media throughout this study, as described previously (Lambert et al. 2005).

We developed primary cultures of bone marrow-derived mast cells (BMMC) from the marrow of the femurs of C57B6 mice, as described by Odom et al. (2004). We obtained wild type (WT) C57B6 mice from the Jackson Laboratory (Houston, TX) and produced ER-α KO mice by back-cross of the previously generated heterozygous ER-α KO mice (Lambert et al. 2005). BMMC cultures contained > 98% pure mast cells after 4 weeks, as assessed by toluidine blue staining. For the last 48 hr before harvesting, we cultured these BMMCs in medium with estrogen-stripped FCS (Invitrogen, Carlsbad, CA). We used BMMCs to confirm that the effects of E_2_ were through ER-α , by comparing the cells from WT and ER-α KO mice. All animal experimental protocols were approved by the University of Texas Medical Branch Institutional Animal Care and Use Committee. The animals were treated humanely and with regard for alleviation of suffering.

### Estrogens

We obtained 17β -estradiol from Sigma-Aldrich Corporation (St. Louis, MO). We used the following environmental estrogens in our studies: organochloride pesticides or their metabolites (endosulfan, dieldrin, and DDE); a by-product of plastics manufacturing (nonylphenol); and the PCBs Aroclor 1242 and Aroclor 1254. We obtained DDE and endosulfan from Ultra Scientific (North Kingstown, RI) and nonylphenol, dieldrin, Aroclor 1242, and Aroclor 1254 from Sigma.

### Patient serum samples

We obtained samples of sera from patients who had a history of asthma and had a positive skin prick test to house dust mites (DM). We complied with all applicable U.S. requirements and/or international regulations (including institutional review board approval), and human participants gave written informed consent prior to the study.

### Mast cell activation experiments

We harvested cells by trypsinization, cultured them on 96-well plates for 2 days to allow membrane receptors to be resynthesized, and then stimulated cells with various concentrations of E_2_ and environmental estrogens for 30 min. To examine the interaction between exposure to environmental estrogens and allergens in the release of allergic mediators, we sensitized BMMC for 1 hr with 100 ng/mL mouse anti-dinitrophenyl (DNP) IgE antibody (Sigma-Aldrich) and HMC-1 cells for 90 min with a 1:5 dilution of patient serum. After washing away unbound IgE, we stimulated cells with DNP-bovine serum albumin (BSA) complexes (10 haptenes per carrier molecule used at 10 ng/mL; Biosearch Technologies, Inc., Novato CA) or 0.75 AU/mL of dust mite allergen extract (*Dermatophagoides farinae*; Hollister-Stier, Spokane, WA) for 30 min in the presence or absence of E_2_ or environmental estrogens. We performed all mediator measurements in duplicate.

### Assessment of degranulation by release of the granular protein β -hex

Enzymatic assays for β -hex have been used extensively to assess the extracellular release of mast cell and basophil granule contents (Dastych et al. 1999). We stimulated cells (2 × 10^4^) in Tyrode’s buffer (Dastych et al. 1999) containing various concentrations of E_2_. We measured β -hex release as previously described (Dastych et al. 1999), using *p*-nitrophenyl-*N*-acetyl-β -D-glucopyranoside (8 mM; Sigma-Aldrich) as the substrate. We expressed the amount of β -hex release into media as the percentage of the total amount of β -hex originally in the cells [% release = 100 × (experimental β -hex release – spontaneous β -hex release) ÷ total cellular β -hex].

### Statistical analyses

**Figure 1:**
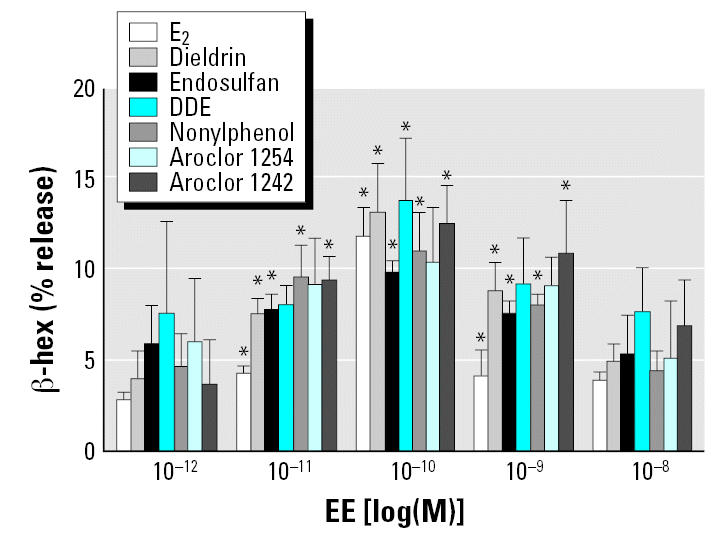
Release of β -hex from HMC-1 after incubation with six different environmental estrogens (EEs): E_2_, dieldrin, endosulfan, DDE, nonylphenol, Aroclor 1254, and Aroclor 1242. Experiments were conducted in triplicate and expressed as mean ± SE. *p < 0.05 vs. phosphate buffered saline control.

**Figure 2:**
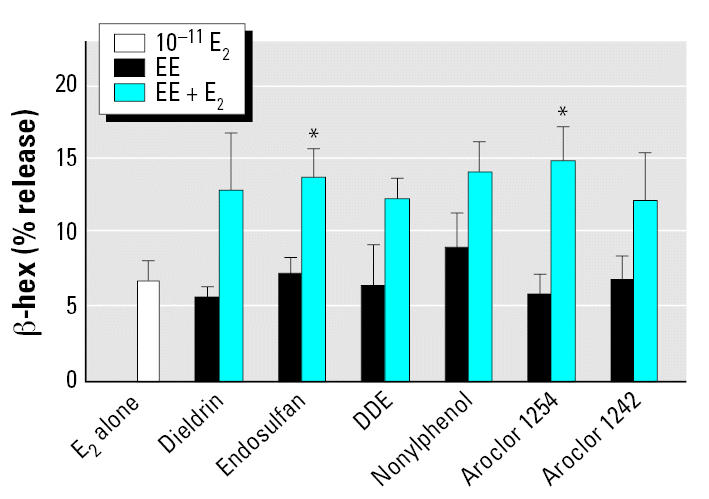
Additive effects of environmental estrogens (EEs) and E_2_ on β -hex release from HMC-1 cells incubated with 10^−11^ M E_2_, dieldrin, endosulfan, DDE, nonylphenol, Aroclor 1254, or Aroclor 1242 alone; or each EE plus E_2_. Experiments were conducted in triplicate and expressed as mean ± SE. *p < 0.05 compared with EE alone.

**Figure 3:**
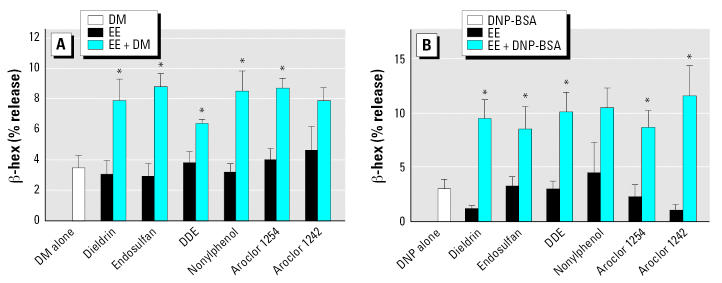
Effects of environmental estrogens (EEs) on IgE-dependent degranulation from HMC-1 cells and BMMC. (*A*) Release of β -hex from HMC-1 cells by 0.75 AU/mL DM alone; 10 pM of dieldrin, endosulfan, DDE, nonylphenol, Aroclor 1254, or Aroclor 1242 alone; or each EE plus DM. (*B*) Release of β -hex from BMMC by anti-DNP IgE/DNP-BSA alone; 10^−13^ M dieldrin, 10^−12^ M endosulfan, 10^−11^ M DDE, 10^−11^ M nonylphenol, 10^−9^ M Aroclor 1254, or 10^−12^ M Aroclor1242 alone; or each EE plus anti-DNP IgE/DNP-BSA. Experiments were conducted in triplicate and expressed as mean ± SE. *p < 0.05 compared with EE alone.

**Figure 4:**
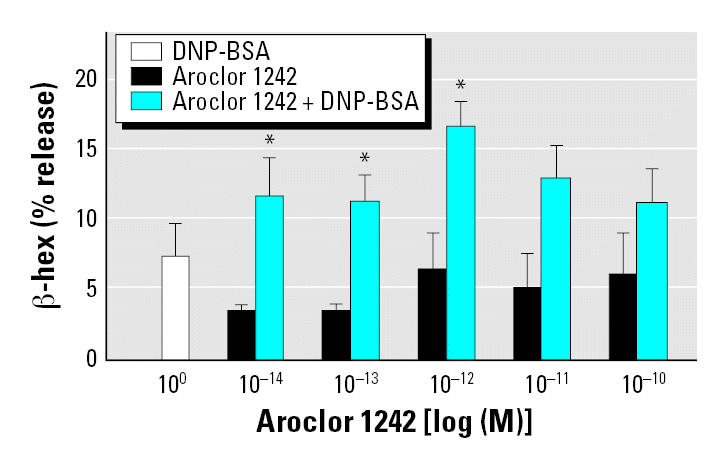
Dose–response effects of DNP-BSA, Aroclor 1242, and Aroclor 1242 plus DNP-BSA on IgE-mediated degranulation of BMMCs from WT mice. Experiments were conducted in triplicate and expressed as mean ± SE. *p < 0.05 compared with Aroclor 1242 alone.

**Figure 5:**
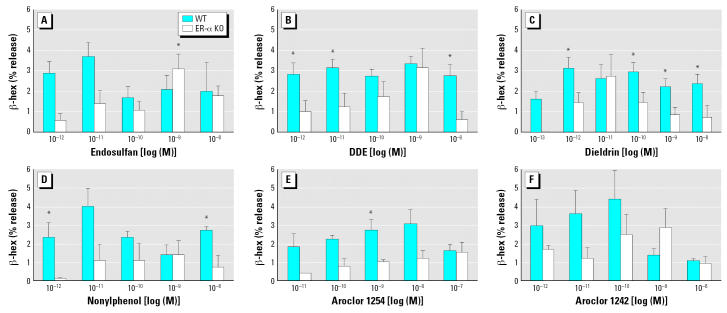
Requirement for ER-α expression for degranulation of BMMCs from WT and ER-α KO mice shown by the release of β -hex by various concentrations of endosulfan (*A*), DDE (*B*), dieldrin (*C*), nonylphenol (*D*), Aroclor 1254 (*E*), and Aroclor 1242 (*F*). Experiments were conducted in triplicate and expressed as mean ± SE. *p < 0.05 WT compared with KO.

Data were expressed as the mean ± SE. Statistical analysis was performed by one-way analysis of variance. Where differences between groups were present, they were further analyzed by the multiple comparisons (Bonferroni) for Figure 1 and Student *t*-test for Figures 2–5. A *p*-value of < 0.05 was defined as statistically significant.

## Results

### Environmental estrogens induce degranulation of HMC-1 cells

We performed a series of experiments to screen for the effects of various concentrations (1 × 10^−12^–10^−8^ M) of E_2_ and six different environmental estrogens on mast cell degranulation, using release of β -hex from HMC-1 cells as a marker for degranulation and release of allergic mediators. Figure 1 shows that all of the environmental estrogens tested except Aroclor 1254 caused the release of a significant portion of intracellular β -hex at concentrations ranging from 10^−11^ to 10^−8^ M after 30 min of stimulation. For comparison, a Ca^2+^ ionophore induced approximately 30% release of intracellular β -hex (data not shown), presumably because not all β -hex resides in releasable granules. Therefore the environmental estrogens alone released up to 50% of the releasable granular contents.

### Combined effects of E_2_ and environmental estrogens on degranulation of HMC-1 cells

To analyze the effect of combinations of endogenous estrogen with environmental estrogens, we incubated HMC-1 cells with combinations of suboptimal concentrations of E_2_ (1 × 10^−11^ M) and varying concentrations of all six estrogenic compounds. We used suboptimal concentrations to test for additive effects, because the release of β -hex from cells incubated with an optimal dose of the estrogenic compounds was not significantly increased by other estrogens (data not shown). Figure 2 shows that these combinations of estrogenic compounds induced degranulation more effectively than either of the compounds alone at these concentrations. The resulting stimulations were approximately additive and again were fairly rapid (< 30 min).

### Environmental estrogens enhance IgE-mediated degranulation of HMC-1 cells and BMMC

We then evaluated the effect of environmental estrogens on IgE-dependent degranulation using our responsive cell systems, which were sensitized with IgE antibodies from the appropriate species. When HMC-1 cells sensitized with human IgE were subsequently exposed to combination of DM allergen and 10^−13^–10^−9^ M environmental estrogens, the release of β -hex was significantly enhanced compared to cells exposed to the same concentration of DM allergen alone (Figure 3A). This was the case for all of the environmental estrogens tested.

We also tested the effects of environmental estrogens on IgE-induced degranulation of primary cultures of BMMCs. We sensitized BMMCs with monoclonal IgE anti-DNP antibodies and stimulated them with DNP-BSA in the presence of 10^−13^–10^−9^ M concentrations of our six test environmental estrogens. Each of these environmental estrogens, except nonylphenol, significantly enhanced the β -hex release induced by DM (Figure 3B). We assessed the dose–response relationship for one of these environmental estrogens (Aroclor 1242) to define the concentrations that had the strongest additive effects on IgE-mediated degranulation and the shape of the dose–response curve. Concentrations of Aroclor 1242 of 10^−14^–10^−12^ M significantly enhanced the effect of IgE cross-linking, whereas higher concentrations of Aroclor 1242 also appeared to increase the response, but not to significant levels (Figure 4).

### ER-α is required for β -hex release induced by some concentrations of environmental estrogens

To determine which types of ERs were involved in the degranulation of mast cells by environmental estrogens, we performed a dose–response analysis on BMMCs derived from WT versus ER-α KO mice. Figure 5 indicates that some concentrations of environmental estrogens induce significantly more degranulation of mast cells from the WT compared with the ER-α KO mice (Figure 5). However, the degranulation response to some concentrations of environmental estrogens was not significantly reduced by the absence of ER-α expression. In fact, many of the concentrations of environmental estrogens alone cause significant degranulation of ER-α –deficient mast cells. This is in contrast to the effects of E_2_, which seems to require ER-α , because E_2_ did not induce significant degranulation from BMMC derived from ER-α KO mice (Zaitsu et al. 2006).

## Discussion

In this study, we examined the effects of environmental estrogens—alone and in combination with physiologic concentrations of E_2_—on the activation of a human mast cell line and primary cultures of murine mast cells. We found that, like E_2_, low concentrations of environmental estrogens caused a rapid, partial degranulation of mast cells. The range of environmental estrogen concentrations that induced β -hex release was somewhat broader for environmental estrogens (10^−8^–10^−12^) compared to that of E_2_ [10^−9^–10^−11^ (Zaitsu et al. 2006)]. However, the dose–response curves for the environmental estrogens were similar to that for E_2_, in that they are biphasic (inverted U-shaped) curves. This type of response is also typical for other steroid-induced responses (Watson et al. 1999; Welshons et al. 2003). Exposing HMC-1 cells to a combination of suboptimal concentrations of E_2_ and an environmental estrogen had an additive effect on degranulation. Environmental estrogens also enhanced the release of β -hex induced by allergen cross-linking of IgE on the surface of these cells. However, when these mast cells were incubated with an optimal dose of environmental estrogens, the addition of E_2_ did not enhance the effects of the environmental estrogen alone (data not shown). Finally, BMMCs deficient in ER-α expression had significantly reduced responses to some concentrations of environmental estrogens, suggesting that at least part of the degranulating activity of environmental estrogens on mast cells is mediated through ER-α .

These findings taken together suggest that the mechanisms of activation of mast cells by environmental estrogens are similar to those of the endogenous estrogen E_2_. Key characteristics of that response are high sensitivity and rapid onset (minutes), partial degranulation, biphasic dose response, requirements for ER-α and extracellular Ca^2+^, and additivity or synergy with IgE cross-linking (Zaitsu et al. 2006). Many of these characteristics are also consistent with those described for activation of the nongenomic (membrane) form of ER-α (Watson et al. 1999; Watson and Gametchu 2003). However, some of the environmental estrogens had residual activity at some concentrations in ER-α KO mast cells. These might be due to compound-specific binding to truncated ER-α in the KO cells (Kos et al. 2002) or to nonclassical ERs, such as the newly described estrogen-binding protein GPR30 (Bologa et al. 2006; Thomas et al. 2005), or other unrecognized receptors.

For instance, we previously described low-dose and rapid effects of environmental estrogens via a membrane-resident ER-α in pituitary tumor cells (Bulayeva and Watson 2004; Wozniak et al. 2005). In that model, environmental estrogens in nanomolar (parts per billion) to picomolar (parts per trillion) concentrations induced extracellular-regulated kinase-1 (ERK-1) and ERK-2 activation via ER-α and Ca^2+^ elevations, leading to rapid prolactin secretion. We have not studied the effects of environmental estrogens on these specific signaling pathways of mast cells, but our recent data suggests that intracellular Ca^2+^ levels rise within 1 min of exposure to E_2_ (Zaitsu et al. 2006).

In the present study, we chose endogenous and environmental estrogen concentrations that would mimic tissue levels that occur in individuals after typical environmental exposures (Ayotte et al. 2003; Ibarluzea et al. 2004; Metcalfe et al. 2001; Solomon and Weiss 2002; Vartiainen et al. 1997; Wang et al. 2004). However, an additional concern is that most environmental estrogens are present in the environment and in tissues and fat stores in combinations, because of their long half-lives and co-prevalence in the environment. Our demonstrations of additive effects between environmental and endogenous estrogens are the first steps toward understanding exposure to complex mixtures of estrogenic compounds. The results of these experiments are consistent with the hypothesis that the effects of both xenoestrogens and physiologic estrogens together will determine the estrogenic impact on an individual. This estrogenic impact is likely to be important both for rapid disease-promoting responses, such as mast cell activation, and for more long-term pathogenesis, such as estrogen-induced cancers.

Some chemicals that accumulate in women’s tissues are also transferred to their infants during breast-feeding. This is especially true for environmental lipid-soluble pollutants such as polyhalogenated compounds, because these chemicals tend to degrade slowly in the environment, to bio-accumulate and bioconcentrate in the food chain, and to have long half-lives in humans. Although the World Health Organization (WHO) strongly supports breast-feeding, breast milk–monitoring studies suggest that environmental chemicals that may affect children’s health are transmitted through breast-feeding (Solomon and Weiss 2002; Wang et al. 2004). Because the fat content of breast milk is relatively high, the concentration of some of these pollutants is 100 times higher in milk than in plasma (Dewailly et al. 1993). As the final consumers in the food chain, human infants may consume the highest concentrations of lipid-soluble environmental pollutants, which might enhance their risk of developing asthma or other allergic diseases.

Our findings on the effects of environmental estrogens on mast cell degranulation may help explain the increasing prevalence of asthma and other allergic diseases in recent decades. A number of sex-steroid effects on immune system functions have been described (Watson and Gametchu 2001), yet relatively few have been explored mechanistically. The results described here indicate that we must also consider the possible impact of environmental estrogens on normal immune function and on the development and morbidity of immunologic diseases such as asthma.
